# Using prescription drug data for timely assessments of state insurance coverage rates: a validation study

**DOI:** 10.1093/haschl/qxag149

**Published:** 2026-06-10

**Authors:** Benjamin N Rome, Adrianna McIntyre, Jinwoo Kim, Jihye Han, Aaron S Kesselheim, Benjamin D Sommers

**Affiliations:** Program on Regulation, Therapeutics, and Law (PORTAL), Division of Pharmacoepidemiology and Pharmacoeconomics, Department of Medicine, Brigham and Women's Hospital and Harvard Medical School, Boston, MA 02115, United States; Department of Health Policy and Management, Harvard T.H. Chan School of Public Health, Boston, MA 02115, United States; Department of Health Policy and Management, Harvard T.H. Chan School of Public Health, Boston, MA 02115, United States; Program on Regulation, Therapeutics, and Law (PORTAL), Division of Pharmacoepidemiology and Pharmacoeconomics, Department of Medicine, Brigham and Women's Hospital and Harvard Medical School, Boston, MA 02115, United States; Department of Health Policy, Vanderbilt University Medical Center, Nashville, TN 37232, United States; Program on Regulation, Therapeutics, and Law (PORTAL), Division of Pharmacoepidemiology and Pharmacoeconomics, Department of Medicine, Brigham and Women's Hospital and Harvard Medical School, Boston, MA 02115, United States; Department of Health Policy and Management, Harvard T.H. Chan School of Public Health, Boston, MA 02115, United States

**Keywords:** Medicaid, uninsured, insurance coverage, prescription drugs, validation study

## Abstract

**Introduction:**

Timely assessments of insurance coverage are limited by lags and unpredictable data availability. We assessed the performance of real-time prescription data as a proxy for state-level coverage changes.

**Methods:**

We analyzed correlations between quarterly state insurance coverage (Medicaid, private insurance, and uninsured) and counts of filled prescriptions per capita by payer (Medicaid, private insurance, and cash-pay/assistance programs) from 2013 to 2024. Regression models measured how predictive prescription counts were of within-state coverage changes.

**Results:**

Medicaid prescriptions per capita were strongly correlated with Medicaid coverage (ρ = 0.62). Cash-pay prescriptions per capita were moderately correlated with uninsured rates (ρ = 0.49). Private coverage correlations were weak. Correlations were stronger for adults (ρ = 0.74 for Medicaid, ρ = 0. 52 for uninsured) than children. Among adults, each 10% within-state increase in Medicaid prescriptions was associated with a 7.5% relative increase (95% confidence interval [CI], 5.7%–9.3%) in Medicaid coverage; each 10% increase in cash-pay prescriptions was associated with a 2.5% increase (95% CI, 1.0%–4.1%) in uninsured rates.

**Conclusions:**

Real-time prescription drug data are moderately to highly correlated with state-level coverage changes in Medicaid and uninsured rates, particularly for adults. These data may be useful for timely assessments of upcoming policies affecting Medicaid coverage.

Key points

- Prescription drug data were strongly correlated with Medicaid enrollment and moderately correlated with uninsured rates, particularly among adults.
- To address lags in the availability of insurance enrollment data, real-time prescription data may be useful for rapid assessments of how upcoming policies affect insurance coverage.

## Background

Adequate health insurance coverage has important implications for health care access and outcomes. Medicaid programs provide an important safety net, and expanded Medicaid coverage under the Affordable Care Act (ACA) was associated with improved health care access, improved patient outcomes, including survival, and reduced health care disparities.^1-4^ The ACA also created Marketplaces for individuals to purchase subsidized private insurance. Together, Medicaid and the Marketplaces covered more than one in four people (27.0%) in the US in 2024.^5^ Largely as a result of these programs, the uninsured rate in the US fell to its lowest level in history in 2023.^6^

The One Big Beautiful Bill Act (OBBBA) passed by Congress and signed into law by President Trump in 2025 included several important provisions expected to affect Americans' access to health insurance, including Medicaid work requirements and more frequent eligibility checks, new restrictions on eligibility for Medicaid and ACA Marketplace subsidies particularly for legal immigrants,^7^ and changes to enrollment processes that will make it more difficult for people to enroll and stay enrolled in both programs. These policies are expected to cause approximately 10 million Americans to become uninsured in the next decade.^8,9^ Additionally, enhanced subsidies for ACA Marketplace plans created during the pandemic expired in the absence of Congressional action in January 2026, leading to substantially higher premium costs for millions of Americans. These higher premiums are projected to lead to another 4 million uninsured individuals.^9,10^

For these policies and other future changes, it will be essential to measure the impact on insurance coverage and health care access. However, timely assessments are often limited by delayed availability of accurate insurance coverage data.^11^ Administrative data on Medicaid enrollment, which depend on reporting by individual states, lag by several months or more. The federal government offers limited administrative Marketplace enrollment figures outside annual open enrollment periods. Data about employer-sponsored insurance, particularly self-employed plans, is even more sparse without a single administrative source. Finally, the gold-standard estimates of state uninsured rates are drawn from federal survey data that lag by a year or more and have been disrupted in recent years by the pandemic, government shutdowns, and changes in data reporting practices under different administrations.^12^

One potential data source that may enable rapid assessment of insurance coverage changes is prescription claims data sourced from retail pharmacies, which are available within days or weeks after prescriptions are filled. Unlike other types of health services, prescription claims are typically adjudicated in real-time, thus avoiding the lags associated with insurance claims data for inpatient or outpatient services. Nearly half of adults in the US take 1 or more prescription medications, meaning that changes in medication capture a large share of the population.^13^ Indeed, prescription drug use data have been previously correlated with changes in insurance coverage following Medicaid expansion^14,15^ and the changes in Medicaid enrollment during and after the COVID-19 pandemic, including the expiration of the continuous coverage provision.^16,17^ These studies have mostly focused on Medicaid coverage. It is less clear whether prescriptions—including cash-pay prescriptions not billed to insurance—are correlated with rates of uninsurance. Furthermore, previous studies have not compared different payers, including private insurance, and have not tested whether associations between drug use and coverage differ by age group and different types of medications.

Real-time prescription claims could serve as a timely proxy to rapidly assess state-based changes in insurance coverage following the implementation of upcoming policy changes. This validation study examined the correlation between state-level quarterly prescription fills per capita and Medicaid, private insurance, and uninsured rates. We also measured the magnitude of the predictive relationship between these measures using multivariable regression models.

## Methods

### Data sources

We compared state-level prescription counts and insurance coverage data from the fourth quarter of 2013 to the end of 2024, a period that included the large coverage expansions under the ACA, the pandemic-era continuous Medicaid coverage provision that prohibited states from disenrolling individuals from Medicaid between March 2020 and March 2023, and the subsequent “unwinding” of that provision in 2023–2024.^17^ The start date of 2013 was chosen because official Medicaid enrollment data were unreliable and not available for all states in prior years. In our primary analysis, we included all states over the entire period. In sensitivity analyses, we stratified states based on whether they ever expanded Medicaid under the ACA between 2014 and 2024, and we stratified the time period as before (2014–2019) and after (2020–2024) the start of the COVID-19 pandemic. The study was determined not to be Human Subjects Research by the Harvard Institutional Review Board. The study was reported in accordance with STROBE guidelines for cross-sectional studies.

This study used prescription fill data from Symphony Health Metys, which estimates national prescription counts based on prescribing data that are collected in real-time from large firms that process most prescription claims for retail, specialty, and mail order pharmacies in the US. This excludes prescriptions for clinician-administered drugs (eg, intravenous infusions) and those from institutional pharmacies (eg, nursing homes, prisons). Data from 2013 to 2024 were stratified by calendar quarter, state, and payer type. Payer types included Medicaid, private insurance, and “cash pay,” which included patients who paid out-of-pocket or with assistance programs like GoodRx. Private insurance included those with employer-sponsored insurance and Marketplace coverage; counts specific to Marketplace plans were unavailable. Data were also stratified by age, separating children (0–18 years) and adults, because the use of medications is different among children and adults. Only one in five children take prescription drugs, and many of these drugs are for acute conditions such as infections, whereas about half of adults aged 45–64 years take prescription medications with a larger share for ongoing conditions.^13,18^ For the primary Medicaid analyses, adults included those aged 19 years and older, because the official Medicaid enrollment files did not distinguish between adults older or younger than 65; for other analyses including privately insured adults, we limited the analysis to those ages 19–64, because nearly all adults 65 and over qualify for Medicare.

We included total prescription counts as well as prescriptions for chronic and acute conditions, separately, using the IQVIA Uniform System of Classification (USC). Chronic conditions included drugs classified as cardiovascular and haematology, diabetes, cancer, psychiatric, and human immunodeficiency virus (HIV), and acute conditions included drugs for cold and allergies and anti-infectives (Appendix Table S1). Prescriptions for these conditions accounted for more than half of all prescriptions, and they include many of the most common chronic conditions in the Medicaid population. These definitions were similar to those used in a prior study of Medicaid prescription drug use.^17^ Separately, we stratified prescription drugs based on whether they were single-source (eg, brand-name drugs that lack generic competition) or multi-source (eg, generic drugs or brand-name drugs for which generic competitors are available); single source drugs tend to be more expensive and thus might be more sensitive to changes in coverage, but multi-source generic drugs make up the majority of prescriptions dispensed in the US.^19^

The primary outcome was total prescription counts, which may have decreased over time as more patients filled 90-day supplies of medications rather than 30-day supplies, particularly following the COVID-19 pandemic.^20^ In a sensitivity analysis, we used adjusted prescription counts holding constant the mean days' supply from the baseline quarter (2014Q1).

We compared prescription drug use with coverage information from several sources. Official Medicaid enrollment statistics were obtained from the Centers for Medicaid and Medicaid Services (CMS).^21^ These data are available monthly, and we averaged them over three months to obtain quarterly estimates. Most states included separate enrollment data for adults (ages ≥19 years) and children (ages 0-18 years); however, some states only reported total enrollment during some years in the study period (Appendix Table S2). Fourteen states had missing periods of missing age-group specific enrollment data of up to 2 years, and these were interpolated based on a linear trend between the values before and after the gaps. Four states had gaps in age-group-specific enrollment data longer than 2 years, and these were treated as missing and excluded from the age-group-based analyses.

The proportions of individuals in each state who were uninsured, or who had private insurance (either employer-based or direct purchase, including Marketplace), were estimated from the US Census Bureau's American Community Survey (ACS).^22^ We similarly stratified coverage by age (0–18 and 19–64 years). We excluded approximately 3% of respondents in the ACS who lived in institutionalized settings, as they would be unlikely to fill medications at pharmacies captured in the Symphony dataset. In a secondary analysis, we also used ACS data to measure the proportion of individuals with Medicaid coverage. We used the administrative data in the primary analysis because prior studies have found that some patients did not realize they had had continuous Medicaid coverage during the COVID-19 pandemic leading to underreporting of Medicaid enrollment during this period.^11,23^ Because of this confusion, however, which research suggests led individuals to lower utilization of care,^24^ it may be the case that prescription drug use tracked more closely with survey-reported coverage than official enrollment statistics. We did not include Medicare coverage in this analysis because eligibility rules for the program do not differ by state, and because the major policy changes in OBBBA and other impending administrative actions are focused on Medicaid and Marketplace eligibility.^12^

To account for differences in state population sizes, all prescription counts and insurance rates were divided by state population for the relevant age group, obtained from the ACS. As a result, insurance coverage was reported as the percentage of individuals covered in each state, and prescription data were reported as the number of prescriptions per capita.

### Statistical analysis

Data were analyzed at the state-quarter level. We first calculated population-weighted pairwise Pearson correlation coefficients between insurance coverage percentage (Medicaid, uninsured, and private) and prescription counts per capita (Medicaid, cash pay, and private). Analyses were conducted overall and by age group (adults and children) and were repeated for prescriptions limited to certain chronic and acute conditions. We also stratified states into two groups based on baseline Medicaid and uninsured rates in 2013; this was done to test whether changes in prescriptions correlated differentially with insurance rates in states with higher or lower coverage. These correlation analyses reflected variation within states (ie, changes over time) and between states, with population weighting to produce nationally representative estimates.

Next, we assessed whether prescription counts per capita were predictive of state coverage rates using a series of ordinary least-squares (OLS) regression models. The dependent variable for each model was the proportion of individuals in the state with a particular type of insurance coverage (Medicaid, uninsured, or private), and the independent variable was the number of prescriptions per capita for the corresponding payer type. The models included fixed effects for each state, which means that the regression coefficients reflected within-state variation in prescription drug use and coverage (ie, changes over time). Both prescription and coverage measures were log-transformed to account for skewness and to provide a ready interpretation: a relative percentage change in coverage as a function of a relative percentage change in prescription counts. Standard errors were clustered by state.

To test the external validity of these regression models, we trained models with the same specifications on data from 2013–2018 and used these models to predict state-quarter coverage in 2019–2024. We measured the correlation between predicted and actual state coverage in each quarter from 2019 to 2024, and the predicted vs actual relative change in coverage in each state over the entire period (2013–2024) and over the period not included in the model (2018–2024).

Results of the correlation and regression analyses were weighted by state population size to yield nationally representative estimates; unweighted correlation coefficients were also reported in a sensitivity analysis. Analyses were performed using Stata version Stata/SE 19.0 (StataCorp, College Station, TX).

## Results

**Figure 1.**
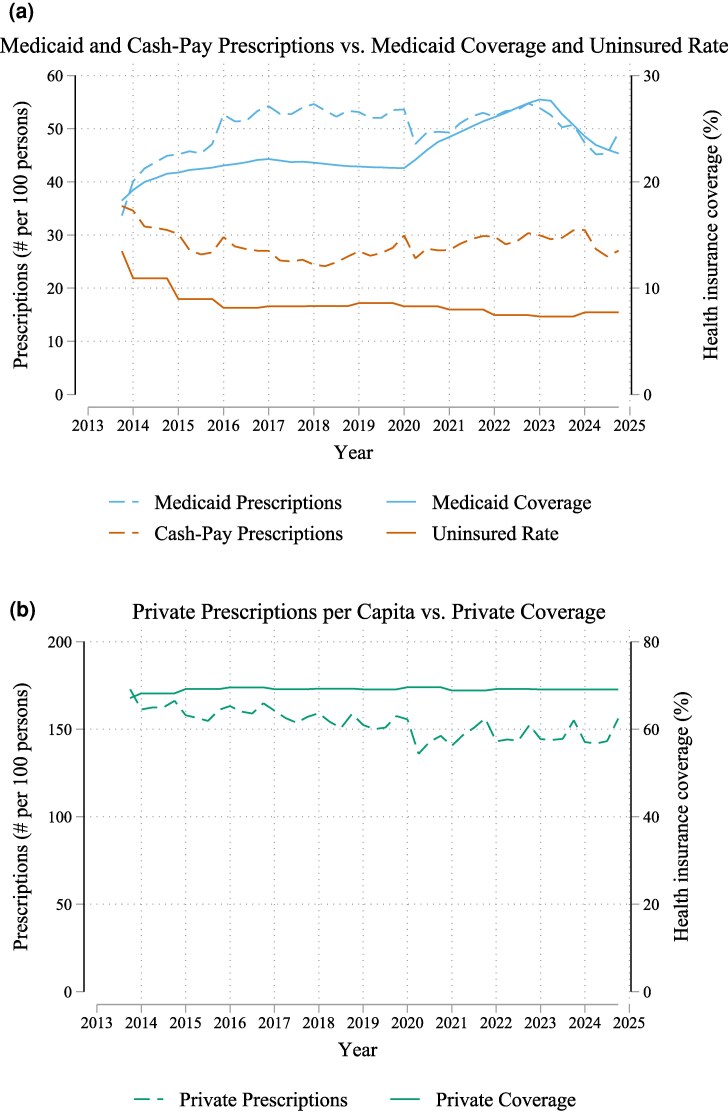
Trends in state prescriptions per capita and insurance enrollment. Self-reported uninsured rates (as a proportion of the population) are from the American Community Survey, and Medicaid enrollment is from the Centers for Medicare & Medicaid Services. Prescription drug counts were from Symphony Health Metys and were reported per capita using data from the American Community Survey. Data represent mean values across all 51 states (including Washington, DC) in each quarter.

The study included a total of 2295 state-quarter observations from 50 states plus Washington, DC. The mean Medicaid coverage rate across the full sample increased from 18.24% in 2013% to 22.67% in 2024, while mean Medicaid prescriptions per 100 persons increased from 33.66 in 2013 to 49.25 in 2024 (Figure 1). In contrast, uninsured rates and cash-pay prescriptions declined over the study period. Private insurance rates were essentially flat, while private prescriptions fluctuated with a slight overall decline during this period. Results were consistent when stratified by age group, although Medicaid prescriptions among children decreased in 2020 (Appendix Figure S1).

**Figure 2.**
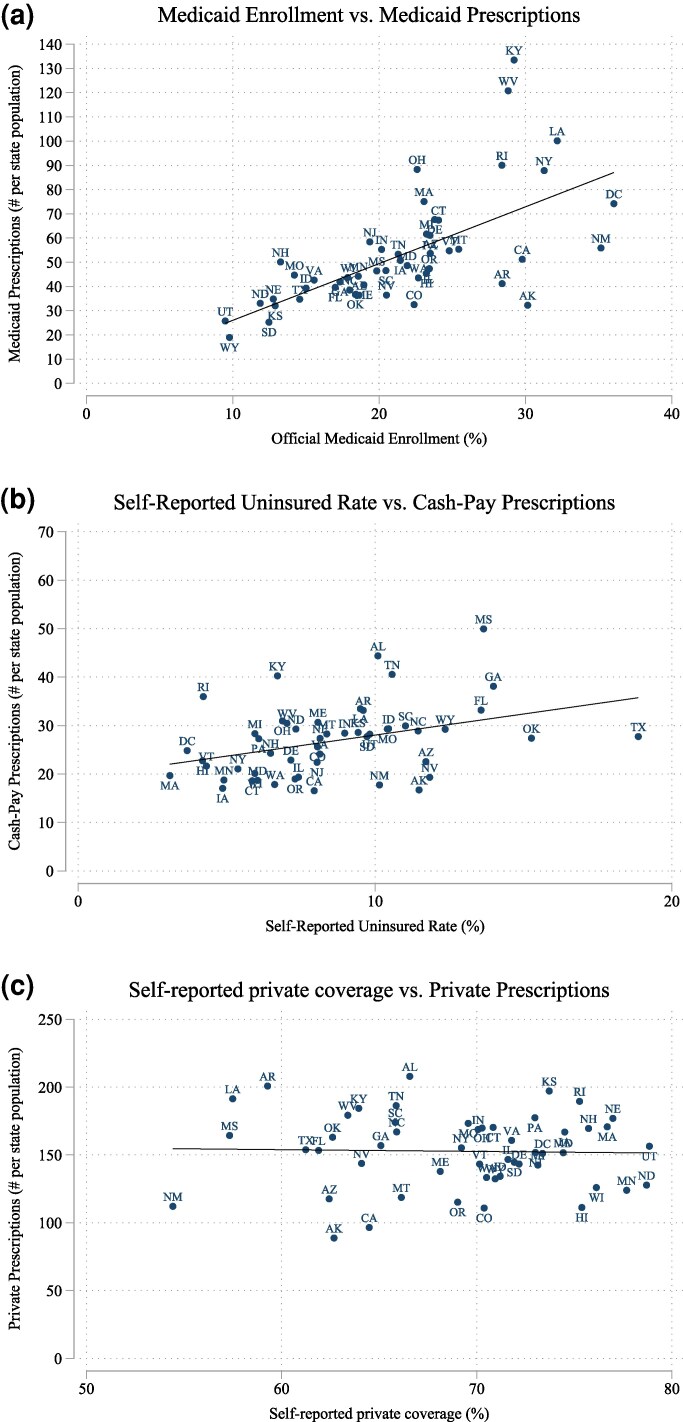
Correlation between state Medicaid enrollment, uninsured rates, and prescriptions per capita, 2019. Sources: State Medicaid Enrollment is from the Centers for Medicare & Medicaid Services and self-reported uninsured rates are from the American Community Survey. Prescription drug counts are from Symphony Health Metys. Both measures were divided by state population counts from the American Community Survey. All prescription values were then multiplied by 100 in the figure. Simple linear regression lines are shown for each plot.

The correlation between state coverage and prescription drug use per capita for 2019 (the last year before the COVID-19 pandemic) is visualized in a scatterplot in Figure 2, demonstrating that Medicaid and uninsured rates were reasonably well correlated with corresponding prescription drug counts between states, while a scatter plot for private coverage rates vs private prescriptions showed no obvious relationship. Results were similar when comparing average results in the pre-pandemic and post-pandemic periods; the association was weaker in the scatter plot for children comparing uninsured rate and cash-pay prescriptions (Appendix Figure S2).

Table 1 shows the correlation results for the full sample and by age group across the full study period, 2013–2024. There was a strong positive correlation (ρ = 0.62; *P* < 0.001) between official state Medicaid enrollment and the number of Medicaid prescriptions per capita; this relationship was stronger for adults (ρ = 0.74; *P* < 0.001) than for children (ρ = 0.38; *P* < 0.001). Results were similar using ACS-reported Medicaid coverage (ρ = 0.63 overall; ρ = 0.79 for adults; ρ = 0.46 for children). There was a moderately positive correlation between uninsured rates and cash-pay prescriptions per capita (ρ = 0.49, *P* < 0.001); this correlation was stronger among adults (ρ = 0.52; *P* < 0.001) than children (ρ = 0.37; *P* < 0.001). The correlation between the rates of private insurance and private insurance prescriptions per capita was weak (ρ = 0.14; *P* < 0.001).

**Table 1.** Correlation between state quarterly filled prescriptions and health insurance coverage rates.

	Medicaid prescriptions per capita vs official Medicaid coverage rate (CMS)	Medicaid prescriptions per capita vs Medicaid coverage Rate (ACS)	Cash-pay prescriptions per capita vs uninsured rate (ACS)	Private prescriptions per capita vs private coverage rate (ACS)^a^
**All ages**	0.62*	0.63*	0.49*	0.14*
**Adults^b^**	0.74*	0.79*	0.52*	0.14*
**Children^c^**	0.38*	0.46*	0.37*	0.18*

**Sources:** Medicaid coverage, self-reported uninsured, and private coverage rates are from the American Community Survey (ACS). Official Medicaid Enrollment is from the Centers for Medicare & Medicaid Services (CMS). Prescription drug counts are from Symphony Health Metys. All coverage and prescription drug data were divided by the state population counts from the ACS. Correlation coefficients were weighted by state population to yield nationally representative estimates.

^a^Includes Non-Group, Marketplace, or Employer Coverage.

^b^Adult Medicaid prescriptions and CMS Medicaid enrollment statistics include individuals aged 19 years or older. ACS Medicaid, cash-pay and private prescriptions, uninsured rate, and private coverage rate are limited to non-elderly adults aged 19-64 years.

^c^Children included individuals aged 0-18 years.

* *P* < 0.001.

For Medicaid, the correlation coefficients were similar when limiting to drugs for chronic or acute conditions (Appendix Table S3). The correlation between cash-pay prescriptions and rates of uninsurance was higher when restricting to drugs for acute conditions, particularly for children (ρ = 0.50 for acute condition drugs vs ρ = 0.37 for all drugs). Correlations were generally lower for all payer types when stratifying medications by single-source vs multi-source (Appendix Table S3) or when examining specific classes of drugs (Appendix Table S4).

Correlation coefficients were similar when stratifying the pre-pandemic (the ACA expansion period) and post-pandemic (continuous coverage and unwinding) periods. Correlations for Medicaid were slightly stronger in non-expansion states (ρ = 0.61) than in expansion states (ρ = 0.53), but this difference disappeared when stratifying by age group (Appendix Table S3). Correlations were also similar among states with low and high baseline Medicaid and uninsured rates (Appendix Table S5). Results were similar without population-weighting (Appendix Table S6) and when adjusting for changes in mean days' supply of prescriptions over time (Appendix Table S7). Additionally, there was a moderately strong negative correlation between Medicaid prescription counts and uninsured rates (ρ = −0.55 overall, and ρ = −0.65 for adults) (Appendix Table S8).

In regression models adjusting for state fixed effects, a 10% relative increase in Medicaid prescriptions per capita was associated with a 4.4% relative increase (95% CI 3.5%–5.5%) in Medicaid enrollment (Table 2; for ease of interpretation here, we multiplied the point estimates in Table 2 by 10, to convey the association with a 10% increase). A 10% increase in cash-pay prescriptions per capita was associated with a 3.6% relative increase (95% CI 2.2%, 4.9%) in self-reported uninsurance rates. In general, these associations were stronger for adults than children. For example, a 10% increase in adult Medicaid prescriptions per capita was associated with a 7.5% increase (95% CI 5.7%, 9.3%) in adult Medicaid enrollment, while for children it was non-significant (−0.2%, 95% CI −0.7%, 0.4%). Meanwhile, regression coefficients for private prescriptions compared to private coverage rates show small relationships close to zero in magnitude (eg, for the total population −0.3%, 95% CI-0.5%, 0.0%). Medicaid associations between prescriptions and coverage were weaker using self-reported Medicaid coverage from the ACS than official Medicaid enrollment.

**Table 2.** Regression coefficients for relative changes in coverage rates vs prescriptions per capita**^a^.**

	Medicaid prescriptions per capita vs official Medicaid coverage rate (CMS)	Medicaid prescriptions per capita vs Medicaid coverage rate (ACS)	Cash-pay prescriptions per capita vs uninsured rate (ACS)	Private prescriptions per capita vs private coverage rate ^b^ (ACS)
**All ages**	0.44*** (0.35, 0.55)	0.28*** (0.22, 0.34)	0.36*** (0.22, 0.49)	−0.03* (−0.05, 0.00)
**Adults ^c^**	0.75*** (0.57, 0.93)	0.54*** (0.44, 0.63)	0.25*** (0.10, 0.41)	−0.09*** (−0.13, −0.06)
**Children ^d^**	−0.02 (−0.07, 0.04)	0.05** (0.01, 0.09)	0.11** (0.02, 0.20)	−0.02* (−0.03, 0.00)

**Sources:** Medicaid coverage, self-reported uninsured, and private coverage rates are from the American Community Survey (ACS). Official Medicaid Enrollment is from the Centers for Medicare & Medicaid Services. Prescription drug counts are from Symphony Health Metys. All coverage and prescription drug data were divided by the corresponding population counts from the ACS.

^a^Each result is from a regression model with an independent variable of log-transformed enrollment rates and a dependent variable of log-transformed prescriptions per capita; all models include state fixed effects and were weighted by state population to yield nationally-representative estimates.

^b^Includes Non-Group, Marketplace, or Employer Coverage.

^c^Adult Medicaid prescriptions and CMS Medicaid enrollment statistics include individuals aged 19 years or older. ACS Medicaid coverage, cash-pay and private prescriptions, uninsured rate, and private coverage rate are limited to non-elderly adults aged 19-64 years.

^d^Children included individuals aged 0-18 years.

^*^** *P* < 0.001, ** *P* < 0.01, * *P* < 0.05.

In models based on 2013–2018 data, predicted and actual state coverage from 2019 to 2023 were highly correlated for Medicaid (ρ = 0.90) and uninsured (ρ = 0.93) (Appendix Table S9). There were also strong correlations between predicted and actual change in Medicaid (ρ = 0.84) and uninsured (ρ = 0.84) rates from 2013 to 2024, and moderate correlations between predicted and actual changes from 2018 to 2024 (Medicaid ρ = 0.55, uninsured ρ = 0.54). Correlations were stronger for adults than for children.

## Discussion

This study of state insurance coverage rates and prescription drug use by payer found that aggregated prescription fill data were strongly correlated with state Medicaid coverage and moderately correlated with state uninsured rates, particularly for adults. Regression-based estimates, which control for state fixed effects and therefore focus specifically on within-state changes in coverage, show significant positive relationships for Medicaid and uninsured rates with prescriptions per capita for those two groups. The results suggest that these prescription data, which are available in real-time and often months to years before gold-standard coverage data, may be an early indication of potential changes in state Medicaid enrollment and uninsured rates as several new policies that limit Medicaid coverage take effect over the coming years.^12^

To put these results in context, a previous analysis comparing various sources of information on health insurance coverage over time and by state found correlation coefficients ranging for 0.47 to 0.59 for state-year coverage rates for Medicaid across federal surveys and a rapid-turnaround telephone survey (in that case, the Gallup-Healthways Well Being Index, which no longer collects health insurance information).^25^ By comparison, the correlation between Medicaid coverage and Medicaid prescriptions per capita in our analysis was slightly stronger, at 0.62. However, the correlation for state-specific uninsured rates and cash-pay prescriptions per capita was weaker in our analysis (0.49) than in that prior analysis comparing Census surveys to the Gallup survey (0.89 to 0.95). By contrast, the correlation for the uninsured rate among non-elderly adults (0.52) was substantially higher than those comparing the uninsured rate between one widely-used federal survey (the Behavioral Risk Factor Surveillance System) and other federal surveys (0.16 to 0.34). Thus, the drug dataset analyzed here performs reasonably well for state Medicaid and uninsured rates compared to a rapid telephone survey used in previous quick-turnaround assessments of policy changes.^26,27^

Our analysis indicates that counts of prescription paid for by private insurance were not a reasonable proxy for private coverage rates, with weak and non-significant correlations and regression-based coefficients close to zero. Although rates of prescription drug use are similar in Medicaid and privately insured populations,^28^ the difference may be that employer-sponsored insurance (ESI) still makes up the vast majority of private insurance in the United States, and rates of ESI have been much more stable over the past decade than have Medicaid and Marketplace insurance.^29^ Thus, policy and economic fluctuations leading to changes in Marketplace insurance may have been imperceptible due to the baseline prevalence of ESI. Alternative prescription drug data sources that distinguish Marketplace coverage from ESI could therefore be a useful area for future research in this regard.

The prescription drug data were weaker predictors of coverage changes among children than adults. This likely reflects two factors. First, prescription drug use among children is much lower on average than among adults and is highly concentrated among children with special health care needs, who are less likely to move in and out of coverage over time.^18,30,31^ Second, the major policy changes affecting coverage over the study period from 2013-2024 had larger effects on eligibility for adults than children, particularly the ACA's Medicaid expansion, meaning there was less meaningful variation over time in children's coverage to identify by examining changes in prescription drug use.

The validity of these results and ability to use changes in prescriptions to predict coverage changes in other contexts was supported by consistent results across several subgroup analyses, including states with and without Medicaid expansion, states with high vs low baseline uninsured rates, and across different years (ACA expansion and pandemic periods). Additionally, in models based on data from the first half of the study period, predicted changes in state Medicaid and uninsured rates during the second half of the period were highly correlated with actual changes, providing additional support that trends in prescription data might be a useful early predictor of coverage changes in response to future policies that differentially affect coverage by state, such as Medicaid work requirements.

There are important limitations in our analysis—and by extension, in future potential analyses using similar drug data to assess coverage impacts. First, prescription drug use is not evenly distributed across the population; about half of adults and 1 in 5 children use prescription drugs, and most prescriptions are concentrated among a small share of individuals.^13,18^ Thus, changes in counts of prescriptions may be sensitive to coverage changes that shift the makeup of the underlying population. Additionally, prescription counts only included outpatient medications dispensed through traditional pharmacies, whereas coverage estimates include institutionalized populations that are not captured in the prescribing data. However, even with these limitations we identified strong within-state and between-state correlation between prescription counts and insurance coverage in several contexts.

Second, the variation in coverage and prescription drug use during our study was context-specific, largely related to the implementation of the ACA in 2014, the pandemic in 2020, enhanced Marketplace subsidies and outreach starting in 2021, and the post-pandemic unwinding period of Medicaid in 2023-2024. Whether the relationships observed here will continue similarly in future policy contexts is unclear. The effects of coverage gains and losses on prescriptions may differ, and the relationship between future coverage losses and prescriptions may be heterogeneous depending on how states respond to federal policy changes. For example, we previously found that states that implemented protective policies to maintain coverage during the post-pandemic Medicaid unwinding had much smaller decreases in prescription drug use, possibly because patients with higher health needs were able to maintain coverage.^17^ If similar policies are implemented to mitigate the effects of Medicaid work requirements and other policy changes, coverage losses may be more concentrated among healthier patients with lower use of prescription drugs, and thus changes in prescription drug use may underestimate coverage losses.^15,32^ It is reassuring that the correlation between prescription drug use and coverage rates were similar in the pre-pandemic (2013-2019) and peri-pandemic (2020-2024) periods.

Finally, other changes in prescription drug use patterns may have influenced our results. Our outcomes were based on the total number of prescriptions filled in each month. Shifts in the length of prescriptions (eg, moving from 30-day to 90-day prescriptions) could create a time-varying source of confounding in our analysis, which would likely bias our correlation coefficients towards zero by introducing measurement error. By 2024, more than half of prescriptions dispensed in the US were for 90-day supplies, a marked increase over the past decase.^20^ However, even when adjusting for this change in days' supply, the correlation between prescription counts and insurance coverage were largely unchanged, suggesting that these shifts did not introduce any state-specific or time-trend bias.

In conclusion, aggregated state-level data on prescription drug use by payer were significantly associated with state-level changes in Medicaid coverage and uninsured rates, particularly among adults. These data may facilitate timely assessments of early coverage changes under new policies while researchers and policymakers await the subsequent release of gold-standard government coverage information.

## Supplementary Material

qxag149_Supplementary_Data
