# Supplementary material for: Using prescription drug data for timely assessments of state insurance coverage rates: a validation study
Source: Health Aff Sch. 2026 Jun 10;4(6):qxag149. doi: 10.1093/haschl/qxag149 (PMC13293373; doi:10.1093/haschl/qxag149)
Supplement: qxag149_Supplementary_Data [file qxag149_supplementary_data.zip › Prescriptions vs coverage Supplement 6-3-2026.docx]

**Using Prescription Drug Data for Timely Assessments of State Insurance Coverage Rates:**

**A Validation Study**

**Supplemental Appendix**

**Table A1.** Definition of Therapeutic Areas in the Study by IQVIA Uniform System of Classification (USC) Codes

**Table A2**. Missing and Imputed State Medicaid Enrollment data

**Figure A1.** Trends in State Prescriptions Per Capita and Insurance Enrollment Stratified by Age Groups, 2013Q4-2024Q4

**Figure A2.** Correlation between state Medicaid enrollment, uninsured rates, and prescriptions per capita

**Table A3.** Correlation Between State Insurance Coverage and Prescriptions per Capita for Subgroups of Prescription Drugs

**Table A4.** Correlation Between State Insurance Coverage and Prescriptions per Capita for Specific Classes of Drugs

**Table A5.** Correlations between Prescription Drug Utilization and Insurance Coverage, by Baseline State Coverage Rates

**Table A6.** Unweighted Correlation Between State Quarterly Filled Prescriptions and Health Insurance Coverage Rate

**Table A7.** Correlation Between Health Insurance Coverage Rates and State Quarterly Filled Prescriptions, Adjusted for Average Days of Supply

**Table A8.** Correlation Matrix for State Quarterly Filled Prescriptions and Health Insurance Coverage Rates

**Table A9.** Correlation Between Predicted and Actual Changes in State Coverage

**Table A1. Definition of Therapeutic Areas in the Study by IQVIA Uniform System of Classification (USC) Codes**

| **Therapeutic Area** | **USC Name** |
| --- | --- |
| Allergy/Cold | - 14000, Allergy/cold preparations - 34000, Cough/cold/flu preparations - 28000, Respiratory Therapy - 28400, Steroids, inhaled - 28420, Steroids, inhaled nasal - 28200, Respiratory NSAID - 28210, Respiratory NSAID, bronchial - 28300, Bronchial Mucolytics - 61000, Ophthalmic Preparations - 61500, Ophthalmic, anti-allergy |
| Anti-infective | - 16000, Antimalarials - 82000, Antivirals (except for HIV, HCV, COVID-19) - 38000, Anti-fungal agents - 15000, Anti-infectives, systemic - 61000, Ophthalmic Preparations - 61100, Ophthalmic anti-infectives - 62000, Otic Preparations - 62300, Otic anti-infectives |
| HIV | - 82000, Antivirals - 82100, HIV antivirals |
| HCV | - 82000, Antivirals - 82200, Non-HIV antivirals - 82210, Viral Hepatis Products - 82212, HCV Antivirals |
| COVID-19 | - 82000, Antivirals - 82200, Non-HIV antivirals - 82250, Coronavirus Antivirals |
| Cancer | - 30000, Antineoplastic chemotherapy - 35000, Antineoplastic targeted therapy |
| Psychiatric | - 64000, Psychotherapeutic drugs |
| Cardiovascular and Hematology | - - - - - 29000, Cardiac agents         - 41000, Diuretics and aquaretics         - 11000, Hemostatic modifiers         - 31000, Vascular agents         - 32000, Antihyperlipidemic agents |
| Diabetes | - - - - - 39000, Diabetes therapy |
| Asthma/COPD | - - - - - 28000, Respiratory Therapy - 28500, Leukotriene agents - 28100, Bronchodilaters, general - 28400, Streroids, inhaled - 28410, Bronchial - 28430, Bronchial combinations - 28900, Respiratory, other |
| Contraceptives | - - - - - 33000, Contraceptives |

**Table A2. Missing and Imputed State Medicaid Enrollment data**

| **State(s)** | **Calendar Quarter(s)** | **Missing Quarter Count** | **Missing Data Type** | **Status** |
| --- | --- | --- | --- | --- |
| **IMPUTED DATA** | | | | |
| Maine | 2014Q1 | 1 | Total enrollment, Age-stratified enrollment | Imputed |
| Connecticut, Iowa, Illinois, Kansas, Nevada | 2014Q1 | 1 | Age-stratified enrollment | Imputed |
| North Dakota | 2014Q1–2014Q2 | 2 | Total enrollment, Age-stratified enrollment | Imputed |
| Arkansas, New Hampshire, Pennsylvania, Rhode Island, Wisconsin | 2014Q1–2014Q2 | 2 | Age-stratified enrollment | Imputed |
| California | 2014Q1–2015Q4 | 8 | Age-stratified enrollment | Imputed |
| Louisiana | 2016Q1 | 1 | Age-stratified enrollment | Imputed |
| **MISSING DATA, Excluded from Analysis** | | | | |
| New Mexico | 2014Q1–2016Q1 | 9 | Age-stratified enrollment | Missing |
| District of Columbia | 2014Q1–2017Q1 | 13 | Age-stratified enrollment | Missing |
| Tennessee | 2014Q1–2019Q1 | 21 | Age-stratified enrollment | Missing |
| Arizona | 2014Q1–2024Q1 | 41 | Age-stratified enrollment | Missing |

Notes:

Official Medicaid Enrollment is from the Centers for Medicare & Medicaid Services. To address missing enrollment data, quarterly values were interpolated between adjacent observed data points, with interpolation limited to gaps of up to two years (≤ 8 consecutive quarters).

**Figure A1. Trends in State Prescriptions Per Capita and Insurance Enrollment Stratified by Age Groups, 2013Q4-2024Q4**

1. **Adult ^a^**

1. **Children ^b^**

**Legend:** Self-reported uninsured rates (as a proportion of the population) are from the American Community Survey, and Medicaid enrollment is from the Centers for Medicare & Medicaid Services. Prescription drug counts were from Symphony Health Metys and were reported per capita using data from the American Community Survey. Data represent mean values across all 51 states (including Washington, DC) in each quarter.

**Notes:**

^a^ Adult Medicaid prescriptions and Medicaid enrollment statistics include 19 years or older. Cash pay and private prescriptions, uninsured rate, and private coverage rate are limited to ages 19-64.

^b^ Children are defined as ages 0-18

**Figure A2. Correlation between state Medicaid enrollment, uninsured rates, and prescriptions per capita**

1. **Medicaid Enrollment vs Medicaid Prescriptions**

1. **Self-Reported Uninsured Rate vs Cash Pay Prescriptions**

**Legend:** State Medicaid Enrollment is from the Centers for Medicare & Medicaid Services and self-reported uninsured rates are from the American Community Survey. Prescription drug counts are from Symphony Health Metys. Both measures were divided by state population counts from the American Community Survey. Adults and children graphs used data from 2019 only. Each dot shows state-level average per-person prescription and health insurance coverage (pre-pandemic: 2014–2019; post-pandemic: 2020–2024). Adult Medicaid prescriptions and Medicaid enrollment statistics include 19 years or older. Cash pay and private prescriptions, uninsured rate, and private coverage rate are limited to ages 19-64. Children are defined as ages 0-18.

Correlations are population-weighted.

**Table A3. Correlation Between State Insurance Coverage and Prescriptions per Capita for Subgroups of Prescription Drugs**

|  | **% of Total Prescriptions Included ^a^** | **Medicaid Prescriptions Per Capita vs. Official Medicaid Coverage Rate** | **Cash-pay Prescriptions Per Capita vs. Uninsured Rate** | **Private Prescriptions Per Capita vs. Private Coverage Rate ^a^** |
| --- | --- | --- | --- | --- |
| **Panel A: All ages** | | | | |
| All medications | 100.00 | 0.62*** | 0.49*** | 0.14*** |
| Chronic disease medications | 40.40 | 0.63*** | 0.41*** | 0.25*** |
| Acute condition medications | 12.61 | 0.44*** | 0.62*** | -0.09*** |
| Single-source medications | 5.56 | 0.51*** | 0.35*** | -0.37*** |
| Multi-source medications | 94.44 | 0.54*** | 0.59*** | -0.49*** |
| Pre-pandemic (2014-2019) | 58.64 | 0.59*** | 0.52*** | 0.15*** |
| Post-pandemic (2020-2024) | 41.36 | 0.70*** | 0.46*** | 0.17*** |
| Medicaid expansion states only | 71.79 | 0.53*** | 0.33*** | 0.34*** |
| Medicaid non-expansion states only | 28.21 | 0.61*** | -0.01 | -0.11** |
| **Panel B: Adults ^c^** | | | | |
| All medications | 100.00 | 0.74*** | 0.52*** | 0.14*** |
| Chronic disease medications | 41.31 | 0.74*** | 0.43*** | 0.32*** |
| Acute condition medications | 11.04 | 0.76*** | 0.60*** | -0.02 |
| Single-source medications | 5.68 | 0.54*** | 0.34*** | -0.46*** |
| Multi-source medications | 94.32 | 0.63*** | 0.60*** | -0.58*** |
| Pre-pandemic (2014-2019) | 58.05 | 0.73*** | 0.53*** | 0.20*** |
| Post-pandemic (2020-2024) | 41.95 | 0.78*** | 0.46*** | 0.25*** |
| Medicaid expansion states only | 72.42 | 0.62*** | 0.29*** | 0.43*** |
| Medicaid non-expansion states only | 27.58 | 0.69*** | 0.06 | -0.08 |
| **Panel C: Children ^d^** | | | | |
| All medications | 100.00 | 0.38*** | 0.37*** | 0.18*** |
| Chronic disease medications | 23.35 | 0.15*** | 0.00 | 0.49*** |
| Acute condition medications | 31.04 | 0.36*** | 0.50*** | -0.14*** |
| Single-source medications | 4.35 | 0.24*** | 0.35*** | -0.32*** |
| Multi-source medications | 95.65 | 0.27*** | 0.53*** | -0.46*** |
| Pre-pandemic (2014-2019) | 61.00 | 0.42*** | 0.37*** | 0.22*** |
| Post-pandemic (2020-2024) | 39.00 | 0.52*** | 0.36*** | 0.20*** |
| Medicaid expansion states only | 68.39 | 0.38*** | 0.22*** | 0.36*** |
| Medicaid non-expansion states only | 31.61 | 0.34*** | 0.07 | -0.19*** |

**Sources:**

Self-reported uninsured and private coverage rates are from the American Community Survey (ACS). Official Medicaid Enrollment is from the Centers for Medicare & Medicaid Services. Prescription drug counts are from Symphony Health Metys. All coverage and prescription drug data were divided by the corresponding population counts from the American Community Survey. Correlation coefficients were weighted by state population to yield nationally-representative estimates.

**Notes:**

^a^ Represents the proportion of all prescriptions from 2013Q4-2024Q4 included in the subgroup.

^b^ Adult Medicaid prescriptions and Medicaid enrollment statistics includes individuals aged 19 years or older. Cash pay and private prescriptions, uninsured rate, and private coverage rate are limited to non-elderly adults aged 19-64 years.

^c^ Children included individuals aged 0-18 years

*** p < 0.001, ** p < 0.01, * p < 0.05.

**Table A4. Correlation Between State Insurance Coverage and Prescriptions per Capita for Specific Classes of Drugs**

|  | **% of Total Prescriptions Included ^a^** | **Medicaid Prescriptions Per Capita vs. Medicaid Coverage Rate** | **Cash-pay Prescriptions Per Capita vs. Uninsured Rate** | **Private Prescriptions Per Capita vs. Private Coverage Rate ^b^** |
| --- | --- | --- | --- | --- |
| **Panel A: All ages** | | | | |
| Allergy/ Cold | 3.24 | 0.40*** | 0.38*** | -0.27*** |
| Anti-Infective | 9.37 | 0.43*** | 0.70*** | -0.02 |
| HIV | 0.24 | 0.47*** | 0.14*** | -0.17*** |
| Cancer | 0.44 | 0.74*** | 0.34*** | 0.26*** |
| Cardiovascular and Hematology | 20.45 | 0.65*** | 0.40*** | 0.13*** |
| Diabetes | 5.20 | 0.78*** | 0.47*** | 0.06*** |
| Psychiatric | 14.07 | 0.41*** | 0.29*** | 0.42*** |
| Asthma/COPD | 4.20 | 0.45*** | 0.40*** | 0.34*** |
| Contraceptives | 2.57 | 0.51*** | 0.50*** | 0.29*** |
| HCV | 0.02 | 0.62*** | 0.12*** | -0.14*** |
| COVID-19 | 0.04 | 0.37*** | -0.09*** | 0.01 |
| **Panel B: Adults ^c^** | | | | |
| Allergy/ Cold | 2.76 | 0.70*** | 0.35*** | -0.16*** |
| Anti-Infective | 8.28 | 0.77*** | 0.70*** | 0.03 |
| HIV | 0.31 | 0.51*** | 0.18*** | -0.32*** |
| Cancer | 0.44 | 0.81*** | 0.31*** | 0.26*** |
| Cardiovascular and Hematology | 20.23 | 0.71*** | 0.42*** | 0.22*** |
| Diabetes | 5.57 | 0.84*** | 0.51*** | 0.12*** |
| Psychiatric | 14.76 | 0.60*** | 0.30*** | 0.45*** |
| Asthma/COPD | 3.52 | 0.65*** | 0.26*** | 0.42*** |
| Contraceptives | 3.05 | 0.61*** | 0.52*** | 0.32*** |
| HCV | 0.02 | 0.68*** | 0.14*** | -0.23*** |
| COVID-19 | 0.04 | 0.36*** | -0.11*** | -0.00 |
| **Panel C: Children ^d^** | | | | |
| Allergy/ Cold | 7.78 | 0.36*** | 0.41*** | -0.48*** |
| Anti-Infective | 23.26 | 0.32*** | 0.47*** | -0.04* |
| HIV | 0.02 | 0.13*** | 0.19*** | 0.13*** |
| Cancer | 0.15 | 0.44*** | 0.00 | 0.40*** |
| Cardiovascular and Hematology | 3.24 | 0.27*** | -0.14*** | 0.30*** |
| Diabetes | 1.31 | 0.45*** | -0.28*** | 0.53*** |
| Psychiatric | 18.62 | 0.10*** | 0.11*** | 0.48*** |
| Asthma/COPD | 10.92 | 0.40*** | 0.56*** | 0.01 |
| Contraceptives | 2.82 | 0.23*** | 0.32*** | 0.32*** |
| HCV | 0.001 | 0.22*** | 0.03 | 0.08*** |
| COVID-19 | 0.01 | 0.27*** | 0.08*** | -0.02 |

**Sources:**

Self-reported uninsured and private coverage rates are from the American Community Survey (ACS). Official Medicaid Enrollment is from the Centers for Medicare & Medicaid Services. Prescription drug counts are from Symphony Health Metys. All coverage and prescription drug data were divided by the corresponding population counts from the American Community Survey. Correlations were weighted by state population to yield nationally-representative estimates.

**Notes:**

^a^ Represents the proportion of all prescriptions from 2013Q4-2024Q4 included in the subgroup.

^b^ Includes Non-Group, Marketplace, or Employer Coverage

^c^ Adult Medicaid prescriptions and Medicaid enrollment statistics include 19 years or older. Cash pay and private prescriptions, uninsured rate, and private coverage rate are limited to ages 19-64.

^d^ Children are defined as ages 0-18

*** p < 0.001, ** p < 0.01, * p < 0.05.

**Table A5. Correlations between Prescription Drug Utilization and Insurance Coverage, by Baseline State Coverage Rates**

|  | **Medicaid Prescriptions Per Capita vs. Official Medicaid Coverage Rate** | | **Cash-pay Prescriptions Per Capita vs. Uninsured Rate** | |
| --- | --- | --- | --- | --- |
|  | States with Below-Median Medicaid Enrollment ^c^ | States with Above-Median Median Enrollment ^c^ | States with Below-Median Uninsured Rates^d^ | States with Above-Median Uninsured Rates^d^ |
| All ages | 0.57*** | 0.50*** | 0.44*** | 0.41*** |
| Adults ^a^ | 0.74*** | 0.65*** | 0.41*** | 0.49*** |
| Children ^b^ | 0.44*** | 0.33*** | 0.14*** | 0.45*** |

**Sources:**

Self-reported uninsured rate is from the American Community Survey (ACS). Official Medicaid Enrollment is from the Centers for Medicare & Medicaid Services. Prescription drug counts are from Symphony Health Metys.

All coverage and prescription drug data were divided by the corresponding population counts from the American Community Survey. Correlations were weighted by state population to yield nationally-representative estimates.

**Notes:**

^a^ Adult Medicaid prescriptions and CMS Medicaid enrollment statistics include individuals aged 19 years or older. Cash-pay prescriptions and uninsured rate are limited to non-elderly adults aged 19–64 years.

^b^ Children included individuals aged 0-18 years

^c^ Defined based on whether a state’s CMS Medicaid/CHIP enrollment rate was below or above the median across states in 2013Q4, the first time period of the study period.

^d^ Defined based on whether a state’s total uninsured rate was below or above the median across states in 2013Q4, the first time period of the study period.

*** p < 0.001, ** p < 0.01, * p < 0.05.

**Table A6. Unweighted Correlation Between State Quarterly Filled Prescriptions and Health Insurance Coverage Rate**

|  | **Medicaid Prescriptions Per Capita vs. Official Medicaid Coverage Rate (CMS; unweighted)** | **Medicaid Prescriptions Per Capita vs. Medicaid Coverage Rate (ACS; unweighted)** | **Cash-pay Prescriptions Per Capita vs. Uninsured Rate (ACS; unweighted)** | **Private Prescriptions Per Capita vs. Private Coverage Rate (ACS; unweighted) ^a^** |
| --- | --- | --- | --- | --- |
| **All ages** | 0.64*** | 0.65*** | 0.44*** | 0.04* |
| **Adults ^b^** | 0.72*** | 0.81*** | 0.48*** | -0.00 |
| **Children ^c^** | 0.60*** | 0.66*** | 0.07*** | 0.17*** |

**Sources:**

Medicaid coverage, self-reported uninsured, and private coverage rates are from the American Community Survey (ACS). Official Medicaid Enrollment is from the Centers for Medicare & Medicaid Services. Prescription drug counts are from Symphony Health Metys. All coverage and prescription drug data were divided by the corresponding population counts from the American Community Survey. This table presents the same analysis as in Table 1, but without population-weighting.

**Notes:**

^a^ Includes Non-Group, Marketplace, or Employer Coverage

^b^ Adult Medicaid prescriptions and CMS Medicaid enrollment statistics include individuals aged 19 years or older. ACS Medicaid, cash-pay and private prescriptions, uninsured rate, and private coverage rate are limited to non-elderly adults aged 19–64 years.

^c^ Children included individuals aged 0-18 years

.*** p < 0.001, ** p < 0.01, * p < 0.05.

**Table A7. Correlation Between Health Insurance Coverage Rates and State Quarterly Filled Prescriptions, Adjusted for Average Days of Supply**

|  | **Medicaid Prescriptions Per Capita, Adjusted for Days of Supply vs. Medicaid Coverage Rate** | **Cash-pay Prescriptions Per Capita, Adjusted for Days of Supply vs. Uninsured Rate** | **Private Prescriptions Per Capita, Adjusted for Days of Supply vs. Private Coverage Rate^a^** |
| --- | --- | --- | --- |
| **All ages** | 0.67*** | 0.48*** | 0.09*** |
| **Adults ^b^** | 0.77*** | 0.57*** | 0.14*** |
| **Children ^c^** | 0.46*** | 0.46*** | 0.20*** |

**Sources:**

Self-reported uninsured and private coverage rates are from the American Community Survey (ACS). Official Medicaid Enrollment is from the Centers for Medicare & Medicaid Services. Prescription drug counts are from Symphony Health Metys. All coverage and prescription drug data were divided by the corresponding population counts from the American Community Survey.

**Notes:**

^a^ Includes Non-Group, Marketplace, or Employer Coverage

^b^ Adult Medicaid prescriptions and Medicaid enrollment statistics include 19 years or older. Cash pay and private prescriptions, uninsured rate, and private coverage rate are limited to ages 19-64.

^c^ Children are defined as ages 0-18

Correlations were weighted by state population to yield nationally-representative estimates.

*** p < 0.001, ** p < 0.01, * p < 0.05.

**Table A8. Correlation Matrix for State Quarterly Filled Prescriptions and Health Insurance Coverage Rates**

|  | **Medicaid Prescriptions Per Capita** | **Cash-pay Prescriptions Per Capita** | **Private Prescriptions Per Capita** |
| --- | --- | --- | --- |
| **Panel A: All ages** | | | |
| Official Medicaid Coverage | 0.62*** | -0.39*** | -0.41*** |
| Self-reported Uninsured | -0.55*** | 0.49*** | 0.13*** |
| Self-reported Private Coverage^a^ | 0.09*** | -0.31*** | 0.14*** |
| **Panel B: Adults^b^** | | | |
| Official Medicaid Coverage | 0.74*** | -0.54*** | -0.46*** |
| Self-reported Uninsured | -0.65*** | 0.52*** | 0.16*** |
| Self-reported Private Coverage^a^ | 0.05** | -0.24*** | 0.14*** |
| **Panel C: Children^c^** | | | |
| Official Medicaid Coverage | 0.38*** | 0.03 | -0.23*** |
| Self-reported Uninsured | 0.19*** | 0.37*** | 0.05** |
| Self-reported Private Coverage^a^ | -0.51*** | -0.28*** | 0.18*** |

**Sources:**

Self-reported uninsured and private coverage rates are from the American Community Survey (ACS). Official Medicaid Enrollment is from the Centers for Medicare & Medicaid Services. Prescription drug counts are from Symphony Health Metys. All coverage and prescription drug data were divided by the corresponding population counts from the American Community Survey.

**Notes:**

^a^ Includes Non-Group, Marketplace, or Employer Coverage

^b^ Adult Medicaid prescriptions and Medicaid enrollment statistics include 19 years or older. Cash pay and private prescriptions, uninsured rate, and private coverage rate are limited to ages 19-64.

^c^ Children are defined as ages 0-18

Correlations were weighted by state population to yield nationally-representative estimates.*** p < 0.001, ** p < 0.01, * p < 0.05.

**Table A9. Correlation Between Predicted and Actual Changes in State Coverage** ^a^

|  | **Official Medicaid**  **Coverage Rate** | **Uninsured Rate** |
| --- | --- | --- |
| **Panel A: All ages** |  |  |
| Coverage rate (2019-2024) ^b^ | 0.90*** | 0.93*** |
| Change in coverage from 2013 to 2024 (actual vs. predicted) ^c^ | 0.84*** | 0.84*** |
| Change in coverage from 2018 to 2024 (actual vs. predicted) ^c^ | 0.55*** | 0.54*** |
| **Panel B: Adults ^d^** |  |  |
| Coverage rate (2019-2024)^b^ | 0.93*** | 0.93*** |
| Change in coverage from 2013 to 2024 (actual vs. predicted) ^c^ | 0.85*** | 0.82*** |
| Change in coverage from 2018 to 2024 (actual vs. predicted) ^d^ | 0.52*** | 0.56*** |
| **Panel C: Children ^e^** |  |  |
| Coverage rate (2019-2024)^b^ | 0.87*** | 0.94*** |
| Change in coverage from 2013 to 2024 (actual vs. predicted) ^c^ | 0.67*** | 0.87*** |
| Change in coverage from 2018 to 2024 (actual vs. predicted) ^c^ | 0.21*** | 0.40*** |

**Sources:**

Official Medicaid Enrollment is from the Centers for Medicare & Medicaid Services. Self-reported uninsured rates are from the American Community Survey (ACS). All coverage and prescription drug data were divided by the corresponding population counts from the American Community Survey.

**Notes:**

^a^ Prediction model trained on 2013–2018 data using state fixed effects and population weights. Correlations were weighted by state population to yield nationally-representative estimates.

^b^ Correlation between predicted and actual coverage rates across all state-quarters from 2019-2024

^c^ Actual change was the difference in mean coverage in 2024 and 2013, and predicted change was the difference between predicted coverage in 2024 and actual baseline coverage in 2013.

^d^ Actual change was the difference in mean coverage in 20218 and 2013, and predicted change was the difference between predicted coverage in 2024 and actual coverage in 2018.

^e^ Children included individuals aged 0-18 years

*** p < 0.001, ** p < 0.01, * p < 0.05.
